# 1189. The Impact of Clinical Staff Pharmacist Driven Weekend Antimicrobial Stewardship Coverage in a Quaternary Hospital in UAE

**DOI:** 10.1093/ofid/ofad500.1029

**Published:** 2023-11-27

**Authors:** Hazem Elrefaei, Osama Al Quteimat, Rami Ismail, Rama Nasef, Mohamed Hisham, Wasim S El Nekidy, Rania ElLababidi

**Affiliations:** Cleveland Clinic Abu Dhabi, Abu Dhabi, Abu Dhabi, United Arab Emirates; Cleveland Clinic Abu Dhabi, Abu Dhabi, Abu Dhabi, United Arab Emirates; Cleveland Clinic Abu Dhabi, Abu Dhabi, Abu Dhabi, United Arab Emirates; Cleveland Clinic Abu Dhabi, Abu Dhabi, Abu Dhabi, United Arab Emirates; Cleveland Clinic Abu Dhabi, Abu Dhabi, Abu Dhabi, United Arab Emirates; Cleveland Clinic Abu Dhabi, Abu Dhabi, Abu Dhabi, United Arab Emirates; Cleveland Clinic Abu Dhabi, Abu Dhabi, Abu Dhabi, United Arab Emirates

## Abstract

**Background:**

Pharmacy antimicrobial stewardship services were extended to cover the weekends, incorporating a clinical staff pharmacist with infectious disease (ID) training to cover antimicrobial stewardship program (ASP) activities on weekends is an opportunity. The aim of this study is to assess the impact of integration ASP clinical staff pharmacist into an ASP on weekends by collecting and analyzing the documented pharmacists’ ASP interventions before and after implantation of the service.

**Methods:**

A single center, pre-post quasi-experimental study. Data were collected retrospectively from the electronic medical record (EMR). The study included at 2 sets of data: pre-implementation (2020) and post-implementation (2021) of an ASP weekend pharmacist,

The primary outcome is to evaluate pharmacist ASP Interventions through prospective audit and feedback review analysis. Secondary outcomes include antibiotics days of therapy (DOT), length of hospital stay (LOS), healthcare associated *Clostridioides difficile* infection (CDI) and infection-related readmission.

**Table 1:**
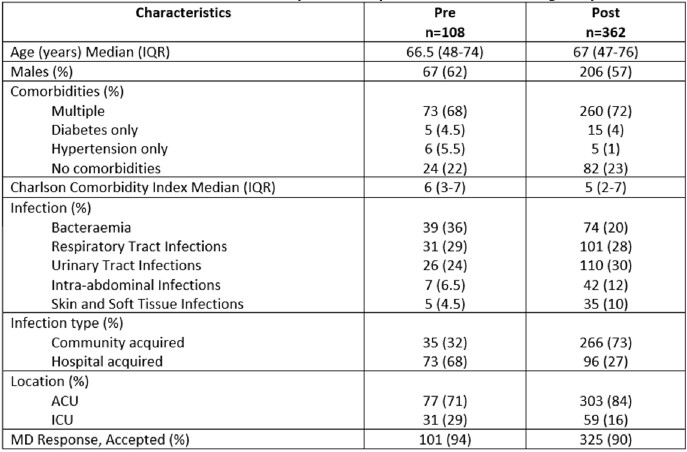
Characteristics of the pre-and post-intervention group

**Results:**

During the study, an increase in the number of documented interventions was observed with 452 interventions were documented on 362 patients during the post-implementation period compared to 114 interventions were documented on 108 patients during the pre-implementation period (p = 0.04).

A reduction in the LOS was observed with a median (IQR) of 16 days (8-34) during the post-implementation period compared to 27.5 days (10-56) during the pre-implementation period (p = 0.001), while the median (IQR) total DOT was increased during the post-implementation period 8 (6-11) versus 7 (4-11) during the pre-implementation period (p = < 0.001). No differences were observed in healthcare associated *Clostridioides difficile* infection (CDI) and infection-related readmission.

**Table 2:**
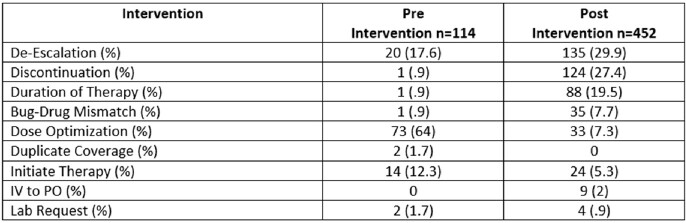
Pharmacist interventions on the pre-and post-intervention group

**Table 3:**
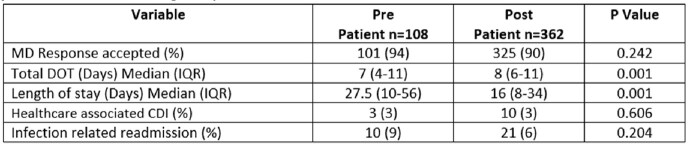
Outcome of pharmacists driven weekend antimicrobial stewardship on the pre-and post-intervention group

**Conclusion:**

The pharmacists driven weekend antimicrobial stewardship is an opportunity for the pharmacists to intervene and optimize patients’ treatment plan and contributed towards a significant shortened length of hospital stay. Therefore, healthcare facilities should prioritize the involvement of pharmacists in weekend ASPs to ensure consistent, high-quality care throughout the week.

**Disclosures:**

**All Authors**: No reported disclosures

